# Management of Complex Duodenal Injuries After Penetrating Trauma

**DOI:** 10.7759/cureus.40431

**Published:** 2023-06-14

**Authors:** Andrew McCague, Brian Patterson, Tracy Taggart

**Affiliations:** 1 Trauma, Desert Regional Medical Center, Palm Springs, USA; 2 Surgery, Desert Regional Medical Center, Palm Springs, USA

**Keywords:** pancreatic injury, trauma, roux-en-y, duodenal injury, gunshot wound

## Abstract

Penetrating injuries to the duodenum can present a complex case for trauma or acute care surgeons. The associated injuries and complications can have devastating results. This report presents the case of a 41-year-old male who presented with a gunshot wound to his abdomen and suffered a gastric injury, transverse colon injury, duodenal injury, renal injury, and pancreatic tail injury. In this case, the patient underwent a complex Roux-en-Y reconstruction. The patient had a good outcome and continues to recover at home.

## Introduction

Penetrating injuries to the duodenum present a difficult case for most trauma surgeons and can lead to devastating complications. This report presents the case of a 41-year-old male who suffered a gunshot wound to his abdomen that injured the fourth portion of his duodenum. The successful intervention is reported, followed by a discussion in this regard.

## Case presentation

A 41-year-old male suffered a gunshot wound to his left upper quadrant abdomen. The patient was initially injured approximately 80 miles from the trauma center. He drove himself to a local police department and was then taken to a local hospital where he was intubated and given two units of packed red blood cells. He was transferred to our trauma center by air. On arrival, his vitals were stable. He was taken to the operating room (OR) urgently for exploration after arrival.

**Figure 1:**
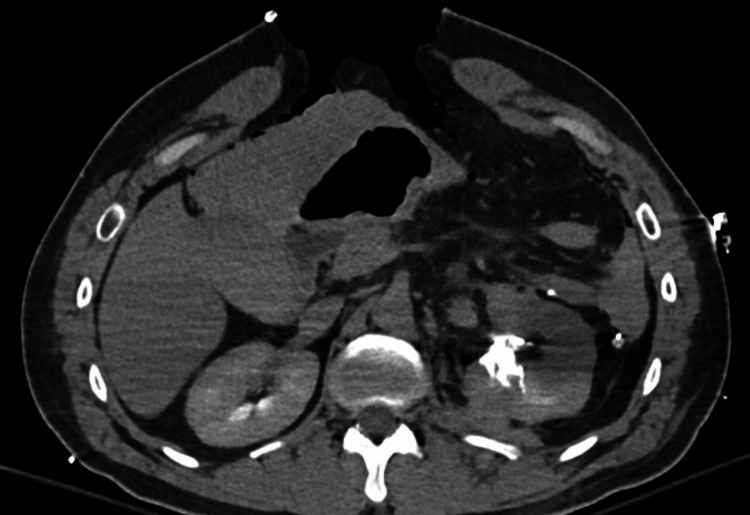
Postoperative CT scan showing a Grade IV left renal injury.

During the intraoperative period, he underwent an exploratory laparotomy where a 1 L hemoperitoneum was encountered. He was found to have two holes in his stomach, a 4 cm defect in the transverse colon, a penetrating injury to the left kidney, and a defect at the fourth portion of the duodenum about 1 cm proximal to the ligament of Treitz. The gastric injuries were closed primarily and sewn over with Lembert sutures. The transverse colon and splenic flexure were mobilized, and the injured area was resected with a linear stapler. The ligament of Treitz was freed to expose the duodenal injury. The injury encompassed 80% of the duodenal circumference. A linear stapler was used to transect the injured portion of the duodenum. A drain was placed over the injured left kidney for possible conservative management. The patient was left in discontinuity with his abdomen left open. He was taken to the intensive care unit (ICU) for continued resuscitation. In the postoperative period, he underwent a computerized tomography (CT) scan, through which postoperative findings and a Grade IV left renal injury were observed (Figure 1).

**Figure 2:**
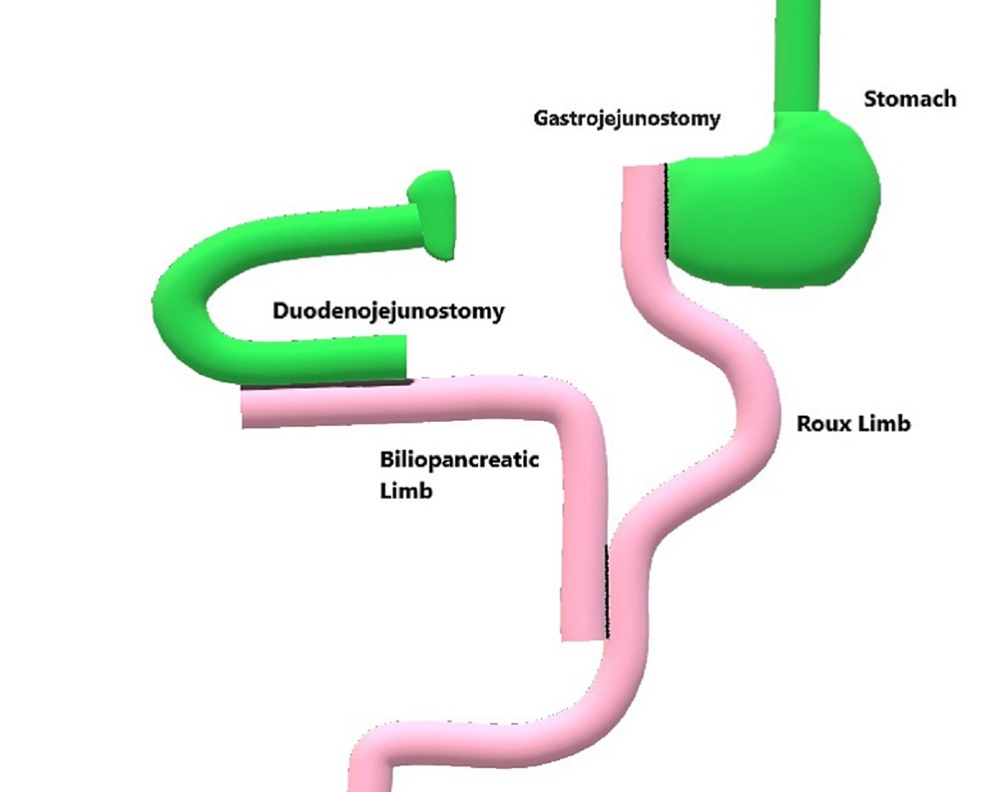
Reconstruction after duodenal injury. The anatomy of the reconstruction is presented in this figure. The stomach can be seen transected with a Roux-en-Y reconstruction, and the duodenojejunostomy and gastrojejunostomy are shown.

The patient was maintained in the ICU for 48 hours where he remained stable after resuscitation. He returned to the OR for re-exploration. The abdomen was explored, and a small area of saponification was found near the tail of the pancreas. The left kidney was found to have a collecting system injury with urine extravasation. Due to concerns of persistent urine leak that could compromise a bowel anastomosis, a left nephrectomy was performed. The duodenum was then examined. A short 1 cm stump at the level of the superior mesenteric artery was found to be pink and viable. A duodenojejunostomy was then planned. To protect from anastomotic or stump leaks, a partial gastrectomy and Roux-en-Y reconstruction were performed. The stomach was transected, and an antrectomy was performed. A Roux limb was brought up retrocolic, and a gastrojejunostomy was performed using linear staplers. The biliopancreatic limb was brought proximal to the duodenum, and a side-to-side duodenojejunostomy was then created using linear staplers. All anastomoses were pink and intact without tension. Two drains were placed: one over the area of nephrectomy and pancreatic tail and another over the gastrojejunostomy and duodenojejunostomy. The fascia was then closed, and the skin was left open. The patient remained intubated and was taken to the ICU for recovery. Figure 2 demonstrates the anatomic representation of the surgical reconstruction.

The patient was extubated the following day - postoperative day (POD) one and post-trauma day (PTD) three. On PTD five, he was transferred from the ICU to the medical-surgical unit. On PTD seven, he was started on a clear liquid diet, and a CT scan was performed that showed a left pleural effusion. A left chest tube was put in place, and it was later removed on PTD 14. His left flank drain fluid displayed elevated amylase, confirming a pancreatic leak. He continued to recover and was later discharged on PTD 21. He underwent a follow-up in the clinic, demonstrating that he has recovered without long-term sequelae.

## Discussion

Duodenal injuries are rare and account for less than 5% of all abdominal injuries caused by trauma [1]. Penetrating injuries are the major cause of duodenal injuries, with gunshot wounds (63.6%) and stab wounds (25%) accounting for most of the injuries [1]. Penetrating duodenal injuries can be devastating and pose a difficult challenge for most trauma surgeons [2]. The duodenum’s proximity to major vascular structures puts the injuries in the area at risk for associated massive hemorrhage and shock [2]. Pancreaticoduodenal injuries are common given the proximity of the duodenum to the pancreas [2]. These patients are at an increased risk of anastomotic leaks and associated morbidity as duodenal repairs have a higher incidence of failure than repairs of other parts of the intestine [2].

While the diagnosis may be straightforward in the case of penetrating trauma, blunt trauma can be more difficult to identify. Even patients taken to the OR for laparotomy may be subject to missed injuries as smaller injuries may be difficult to identify. In particular, blunt trauma, when diagnosed intraoperatively or with a CT scan, may predispose a patient to a missed or delayed injury that can complicate recovery.

Methods to manage duodenal injuries include early exploration with primary closure when possible and wide drainage of the anastomotic site. A more complex procedure is required in more complex injuries or in cases that necessitate the potential healing of an anastomotic site. In this regard, Roux-en-Y reconstruction, decompressive tube duodenostomy placement, and even pyloric exclusion are techniques often used to manage such injuries. Furthermore, severe injuries with uncontrollable hemorrhage or devascularization of the pancreatic head and duodenum may require pancreaticoduodenectomy.

Patients who suffer duodenal injuries often experience complicated postoperative courses. As such, rapid resuscitation, aggressive treatment of shock, and early recognition of any complications are critical to survival. Antibiotics are recommended and should be continued postoperatively; however, the duration of treatment is less clear. These patients often need prolonged bowel rest and early nutritional support, including enteral or parenteral nutrition [1].

Complications are common, occurring in 61% of all patients that survive beyond the first 24 hours. Duodenal injuries are associated with injuries to other nearby structures, including the liver (55%), stomach (46%), and colon (32%). Overall, 31% of patients develop intra-abdominal abscesses, and this emphasizes the need for optimal control of contamination and removal of devitalized tissue. Reports have demonstrated that intra-abdominal abscesses occur most frequently with co-existing colonic injuries [1].

Management recommendations for duodenal injuries are based on the location and injury classification, as described in Table 1.

**Table 1:** Duodenal injury scale and management *Table adapted from the American Association for the Surgery of Trauma (AAST) Duodenal Injury Scale [2].

Grade	Description	Management
I	Partial without perforation	Primary closure
II	<50% circumferential disruption	Tension-free repair is preferred
III	2nd portion, 50–75% disruption 1st, 3rd, 4th portions, with 75–100% disruption	Tension-free primary repair. If A is not possible, a limb of the jejunum is brought up to the defect, performing a Roux-en-Y duodenojejunostomy. If the injury is proximal to the ampulla, close the distal duodenum and perform either a Roux-en-Y duodenojejunostomy to the proximal end OR an antrectomy with gastrojejunostomy.
IVa	2nd portion, >75% circumferential	Tension-free primary repair. If A is not possible, a limb of the jejunum is brought up to the defect, performing a Roux-en-Y duodenojejunostomy. If the injury is proximal to the ampulla, close the distal duodenum and perform either a Roux-en-Y duodenojejunostomy to the proximal end OR an antrectomy with gastrojejunostomy.
IVb	Injury including the ampulla or distal common bile duct	Complex reconstruction with Roux-en-Y limb or pancreaticoduodenectomy
V	Mass destruction of the duodenopancreatic complex	Complex reconstruction with Roux-en-Y limb or pancreaticoduodenectomy

While the initial phase is often managed by trauma or acute care surgeons, the later reconstructive phase often includes hepatobiliary surgeons when available [3]. Patients needing pancreaticoduodenectomy are best managed at facilities that perform pancreaticoduodenectomy frequently to optimize care. Unstable patients with evidence of physiologic compromise, including acidosis, coagulopathy, or hypothermia, may need a damage-control approach [2]. The decision to proceed with a damage-control approach should be made early in the patient’s operative care [2].

Duodenal hematomas are not uncommon and are often diagnosed via a CT scan or intraoperatively during laparotomy. They are typically managed through nasogastric tube decompression and bowel rest. If the obstruction is not resolved within 14 days, then surgical intervention is necessary to drain the hematoma and repair the injury. When diagnosed during laparotomy, the injury should be repaired primarily if possible [2].

The repair of duodenal injuries is based on their grade (see Table 1). Grade I injuries are usually managed with primary repair and postoperative bowel rest. Grade II lacerations are managed with a simple, tension-free repair. A transverse orientation is preferred to prevent stricture. If tension-free repair is not possible and the defect is less than 50% duodenal circumference, the edges of the duodenal injury should be debrided back to healthy tissues, and a limb of the jejunum should be brought up to the defect to perform a Roux-en-Y duodenojejunostomy. For more extensive injuries, the duodenum can be closed, and a Roux-en-Y duodenojejunostomy can be performed to a proximal portion of the duodenum. If the injury is at the first or second portion of the duodenum, another option is to close the distal duodenum (containing the ampulla), perform a formal antrectomy, and reconstruct with a gastrojejunostomy [2].

Grade V injuries are often devastating. These patients present in shock and often require damage-control surgery. If the patient survives to undergo reconstructive surgery, complex repairs and/or resections may be necessary. If the duodenum can be repaired using reconstructive techniques as described above, then the bile duct may be replanted into the duodenum or anastomosed to a Roux-en-Y jejunal loop. If the duodenum cannot be repaired and/or the pancreatic head is injured, a pancreaticoduodenectomy may be indicated [2].

Pyloric exclusion was initially described at the Ben Taub Hospital in Houston, USA. This procedure involves primarily repairing the duodenum, closing the pylorus from within through a gastrostomy, and finally performing a gastrojejunostomy at the site of the gastrotomy. The need for gastrojejunostomy is less clear as recent literature has suggested that the pylorus spontaneously opens within three weeks in the majority of patients. Ulceration at the gastrojejunostomy is not an uncommon complication. The use of pyloric exclusion has become less common in recent years, and today, its use is becoming increasingly controversial [2].

Another option to consider in the management of duodenal injuries is tube duodenostomy. In this procedure, a tube is placed at either the lateral side or the end of the duodenum near the site of the injury. Surgeons often have concerns about an increased risk of leakage from tube duodenostomy; however, other options for reconstruction may not be possible [2].

In the case described above, the patient sustained multisystem trauma. Postoperative complications posed a significant concern in this case, including the risk of anastomotic leaks or stump blowouts. It was felt that proceeding with a Roux-en-Y duodenojejunostomy along with an antrectomy and a gastrojejunostomy would not only protect the anastomosis but also allow for feeding in the case of an anastomotic leak or stump blowout. It also left open the option of additional revision, including resecting back to the third portion of the duodenum or even performing tube duodenostomy if complications were to arise. In this case, the patient was fortunate in that the anastomosis and stump were maintained, and he was successfully discharged.

Ultimately, these cases pose challenges for most trauma and acute care surgeons, with each patient needing an approach tailored to their injuries and clinical presentation.

## Conclusions

Penetrating duodenal trauma presents a complex case for most surgeons. Initial management is often led by a trauma or acute care surgeon who is first available upon the patient’s arrival. There may be limited time to bring in hepatobiliary or other specialists to assist. Early control of hemorrhage and contamination is key to survival. Primary repair may be possible when a tension-free repair can be achieved. Complex reconstruction options are needed for higher-grade injuries. The presented case illustrates an example of a high-grade duodenal injury needing complex reconstruction.
